# A novel *Streptomyces* species producing thiolutin with anti-MRSA activity and insights into its biosynthetic gene cluster

**DOI:** 10.3389/fmicb.2026.1759196

**Published:** 2026-02-27

**Authors:** Xiaomin Niu, Yujie Wu, Xue Yu, Shiyu Wu, Ying Wen, Gaosen Zhang, Guangxiu Liu, Wei Zhang, Kan Jiang, Hang Yang, Mingyue Zhong, Jingjing Jiang, Haitao Yu

**Affiliations:** 1 State Key Laboratory of Ecological Safety and Sustainable Development in Arid Lands, Northwest Institute of Eco-Environment and Resources, Chinese Academy of Sciences, Lanzhou, China; 2 Key Laboratory of Extreme Environmental Microbial Resources and Engineering of Gansu Province, Northwest Institute of Eco-Environment and Resources, Chinese Academy of Sciences, Lanzhou, China; 3 University of Chinese Academy of Sciences, Beijing, China; 4 State Key Laboratory of Cryospheric Science and Frozen Soil Engineering, Northwest Institute of Eco-Environment and Resources, Chinese Academy of Sciences, Lanzhou, China; 5 State Key Laboratory of Aridland Crop Science, College of Agronomy, Gansu Agricultural University, Lanzhou, Gansu, China; 6 State Key Laboratory of Virology and Biosafety, Wuhan Institute of Virology, Chinese Academy of Sciences, Wuhan, China; 7 Hubei Jiangxia Laboratory, Wuhan, China; 8 Institute of Plant Protection, Gansu Academy of Agricultural Sciences, Lanzhou, China

**Keywords:** anti-MRSA activity, biosynthetic pathway, desert *Streptomyces*, polyphasic taxonomic characterization, thiolutin

## Abstract

Exploring microbial diversity from extreme environments, combined with targeted strategies, is a promising approach to uncover novel antimicrobial agents. In order to discover compounds with antimicrobial activity, the sulfur-containing antibiotic thiolutin was isolated and identified from strain HMX112*^T^* using a targeted antibacterial screening strategy. Strain HMX112*^T^* was isolated from a soil sample collected from the Turpan-Hami Basin in China and was characterized as an actinobacterium. 16S rRNA gene sequence analysis revealed that strain HMX112*^T^* showed the highest similarity to *Streptomyces lateritius* CGMCC 4.1427*^T^*, *Streptomyces narbonensis* CGMCC 4.1737*^T^*, and *Streptomyces purpureus* DSM 43362*^T^*, with sequence similarities of 98.83, 98.83, and 98.76%, respectively. Whole-genome analysis indicated that the average nucleotide identity (ANI) and digital DNA–DNA hybridization (dDDH) values between strain HMX112*^T^* and its closest related type strains were well below the thresholds for novel species delineation (95 and 70%, respectively). Based on polyphasic taxonomic analysis, strain HMX112*^T^* was confirmed to represent a novel species of the genus *Streptomyces*, for which the name *Streptomyces flavimicrosus* sp. *nov.* was proposed. Based on its observed antibacterial activity against Gram-positive bacteria, the bioactive components of strain HMX112*^T^* were investigated, resulting in the isolation and identification of the secondary metabolite thiolutin. Antimicrobial assays demonstrated that thiolutin possesses broad-spectrum inhibitory activity, showing MIC values of 16 μg/mL against two MRSA strains and inhibition rates exceeding 70% against nine plant pathogenic fungi at a concentration of 50 μg/mL. Furthermore, the biosynthetic pathway of thiolutin was elucidated through genomic analysis.

## Introduction

Between 1990 and 2021, antimicrobial resistance (AMR) was associated with over one million deaths annually, a figure projected to rise to nearly two million by 2050 (Naghavi et al., 2024). Among these, infections caused by methicillin-resistant *Staphylococcus aureus* (MRSA) have contributed to the largest increase in mortality, with deaths attributable to MRSA rising by 90% (Naghavi et al., 2024). This escalating public health threat underscores the urgent need for novel bioactive compounds to combat drug-resistant pathogens and emerging infectious diseases (Choudhary et al., 2016). Microbial natural products represent a vital source of such bioactive agents, with derivatives from *Streptomyces* alone accounting for more than 70% of clinically used antibiotics (Traxler and Kolter, 2015). These natural products (NPs) are secondary metabolites biosynthesized by bacteria and fungi. Although non-essential for basic microbial growth and development, they often play roles in defense, regulation, and cell-to-cell signaling (Traxler and Kolter, 2015). Since the discovery of penicillin, over 23,000 NPs have been identified from microbial (Katz and Baltz, 2016). However, with the increasing exploration of microbial metabolites, the pace of compound rediscovery has gradually slowed. Therefore, isolating novel microbial species from extreme habitats and exploring their NPs potential remains a highly valuable strategy for discovering new chemical entities.

Deserts impose severe challenges on microbial survival due to their extreme aridity, intense solar radiation, drastic temperature fluctuations, and severe nutrient limitation (Horikoshi et al., 2010, Köberl et al., 2011). To cope with these environmental stresses and to compete for survival, desert microorganisms have likely evolved unique secondary metabolic pathways and novel regulatory mechanisms during long-term evolution, making them a rich reservoir for the discovery of new natural products with distinct bioactivities (Xie and Pathom-Aree, 2021). Actinomycetes represent dominant and widely distributed microbial taxa in desert ecosystems (Wen et al., 2023). Compared with ordinary soils or other extreme habitats, desert soils harbor a higher abundance of *Streptomyces*, with proportions exceeding 50% in some arid regions, suggesting a high potential for the presence of rare and previously uncharacterized *Streptomyces* taxa (Kemung et al., 2018, Sivalingam et al., 2019). A large number of novel actinomycete species, such as *Streptomyces leeuwenhoekii* and Amycolatopsis desertii, have been isolated from the Atacama Desert (Bull et al., 2016, Goodfellow et al., 2018). These microorganisms have subsequently been shown to produce a diverse array of structurally novel and biologically active natural products, including ansamycins (e.g., chaxamycins), macrolides (e.g., chaxalactins and atacamamycins), aminobenzoquinones (abenquines), and ribosomally synthesized and post-translationally modified peptides (RiPPs, such as chaxapeptin), many of which exhibit potent activities against Gram-positive bacteria and tumor cells (Rateb et al., 2011, Abdelkader et al., 2018). As a highly promising yet underexplored biological resource, desert-derived actinomycetes in China are increasingly recognized for their immense potential in the discovery of novel bioactive natural products.

Among diverse NPs, those with unique structures are instrumental in identifying valuable antimicrobial targets, thereby informing the design of novel antibiotics. Notably, the disulfide pyrrolones represent a class of antibiotics with broad spectrum activity against Gram-positive and Gram-negative bacteria and fungi. Notably, it is effective against resistant pathogens such as MRSA, *Klebsiella pneumoniae*, and *Pseudomonas aeruginosa* (Li et al., 2014). Furthermore, disulfide pyrrolones, including thiolutin, have recently demonstrated antitumor potential. Despite its promising bioactivity, the limited natural availability of thiolutin and the incomplete understanding of its biosynthesis and regulation across different microbial strains present significant challenges to its further development and application.

While the biosynthetic pathways of dithiolopyrrolone compounds have been partially elucidated, the specific route for thiolutin production remains incompletely understood. To address this gap, we conducted a screen of desert-derived microorganisms from China and identified strain HMX112 based on its potent anti-MRSA activity. This study reports the discovery of this novel bioactive strain and sets out to achieve three primary objectives: to isolate and characterize the strain as a new *Streptomyces* species, to isolate and identify thiolutin as its active metabolite, and to investigate the genomic basis and biosynthetic gene cluster responsible for thiolutin production.

## Materials and methods

### Sampling and cultivation and phylogenetic analysis of 16s rRNA

Strain HMX112 was isolated from a desert soil sample collected from the Turpan-Hami Basin in China (43°23’53.45”N, 91°48’56.84”E, 1183.49 m). Approximately 5 g of the soil sample was dissolved in 25 mL of sterile saline solution. The suspension was then serially diluted with phosphate-buffered saline (PBS). Aliquots (100 μL) of the dilutions were spread onto Gauze’s No. 1 agar plates and incubated aerobically at 30°C. After incubation, individual colonies with diverse morphologies were selected and purified by repeated streaking on Gauze’s No. 1 medium at 30°C. The purified cultures were preserved at -80°C for long-term storage. Subsequently, strain HMX112 was cultivated again on Gauze’s No. 1 agar to provide biomass for subsequent novel species identification and genetic analysis.

Genomic DNA was extracted using the Wizard Genomic DNA Purification Kit (Promega) according to the manufacturer’s instructions. The 16S rRNA gene was amplified by PCR following a previously described method (Xie and Pathom-Aree, 2021). The PCR products were purified with the E.Z.N.A.^®^ Gel Extraction Kit (Omega Bio-Tek) and subjected to bidirectional sequencing by Sangon Biotech (Shanghai, China). The obtained 16S rRNA gene sequence was analyzed for similarity against related type strains using the EzBioCloud^1^ (Bi and Yu, 2016) and subsequently deposited into the GenBank database. A phylogenetic tree was reconstructed with MEGA 7.0 software (Okoro et al., 2009) based on the most closely related sequences. For a comprehensive taxonomic comparison, the three most phylogenetically related *Streptomyces* type strains were obtained from the China General Microbiological Culture Collection Center (CGMCC) and the German Collection of Microorganisms and Cell Cultures (DSMZ). A series of comparative experiments were then conducted between these reference strains and strain HMX112.

### Phenotypic and biochemical tests of strain HMX112*^T^*

To characterize the phenotypic properties of strain HMX112*^T^*, it was cultivated on Gauze’s No. 1 agar medium, and its spore morphology was examined using scanning electron microscopy (SEM) after fixation with glutaraldehyde, dehydration through an ethanol series, critical-point drying, and gold coating, as described previously (Mohammed and Abdullah, 2018). A broad range of phenotypic traits were assessed using the Biolog GEN III MicroPlate system, in addition to conventional biochemical, physiological, and growth assays relevant to the genus *Streptomyces*. All phenotypic data were obtained from two or three independent replicates using commercial assay kits. Cellular fatty acids were extracted, methylated, and analyzed using the Microbial Identification System (MIDI) version 6.0 (Sasser, 1990). Polar lipids were separated by two-dimensional thin-layer chromatography (TLC) on silica gel plates and identified using specific spray reagents according to the method of Minnikin et al. (1977). The composition of cell-wall diaminopimelic acid and whole-cell sugars was determined by TLC following established protocols (Staneck and Roberts, 1974). Menaquinones were extracted from freeze-dried biomass and purified using the method outlined by Collins (1985).

### Genome sequencing and analysis

Genomic DNA was extracted using the Wizard Genomic DNA Purification Kit and was quantified with a TBS-380 fluorometer (Turner BioSystems). High-quality DNA (OD_260_/_280_ = 1.8–2.0, total > 20 μg) was used for subsequent analysis. Genome sequencing of strain HMX112^T^ was performed using both PacBio RS II and Illumina HiSeq 2000 platforms at Majorbio Bio-Pharm Technology Co., Ltd. (Shanghai, China). A high-quality dataset was generated with approximately 100 × coverage. Raw reads were preprocessed and filtered using the Illumina analysis pipeline (Toh et al., 2017). *De novo* assembly was carried out using the Celera Assembler (version 8.0) (Loman et al., 2015), resulting in contigs that were further scaffolded into two final scaffolds. Gene prediction was performed using Glimmer (Delcher et al., 2007), GeneMarkS (Besemer and Borodovsky, 2005), and Prodigal (Hyatt et al., 2010). Predicted genes were functionally classified and assigned to metabolic pathways using BLASTP (Samal et al., 2021) against the eggNOG and CARD (Comprehensive Antibiotic Research Database) databases. A circular genome map was generated with Circos (Krzywinski et al., 2009). Pan-genome analysis was performed using the Or-thoVenn3 web platform^2^ with default parameters (Sun et al., 2023). TheANI was computed using the ANI calculator available on the EzBioCloud platform (Yoon et al., 2017). Biosynthetic gene clusters (BGCs) for secondary metabolites were predicted with antiSMASH 8.0 (Blin et al., 2025). The identification and genomic localization of 16S rRNA genes were determined using Barrnap. To thoroughly analyze the evolutionary origins of the target biosynthetic gene cluster, BLASTX analysis was performed on all its encoded genes (36 in total). Using the NCBI BLASTX program against the non-redundant protein database (nr), the best-matching sequence, source species, percentage identity, and E-value for each gene-encoded protein were obtained (Wang and Gribskov, 2025).

### Antibacterial activity assay

The antimicrobial activity was evaluated using standard methodologies against diverse pathogens. For common pathogenic bacteria, the agar diffusion method was employed: bacterial suspensions (1 × 10^8^–1 × 10^9^ CFU/mL) were mixed with molten LB agar (2% v/v) and poured into plates. Subsequently, 6 mm wells were created and filled with 20 μL of test samples. After 8 h of incubation at 37°C, the inhibition zone diameters were measured (Wang, 2005). For MRSA strains, a microbroth dilution method was conducted in 96-well plates, with vancomycin as a positive control and solvent as a negative control. The minimum inhibitory concentration (MIC) was determined based on OD_600_ measurements and a standard curve (Alexander et al., 2017). Antifungal activity against plant pathogenic fungi was assessed using the mycelial growth rate method (Shentu et al., 2020). Mycelial plugs (6 mm diameter) from 5 day old cultures were transferred to solid medium containing the test sample, and after incubation at 28°C, the inhibition rate of mycelial growth was calculated relative to solvent-treated controls. All experiments were performed in triplicate.

### General chemical experimental procedures

The crude fermentation extract was initially fractionated using normal-phase column chromatography packed with 200–300 mesh silica gel, with TLC for monitoring. The separation was performed with a gradient elution of petroleum ether, ethyl acetate, and acetone. Further analysis was carried out on an Elite EClassical 3100 HPLC system equipped with a C18 analytical column (SinoChrom ODS-BP, 4.6 × 250 mm). The injection volume was 20 μL, and detection was achieved with a UV detector set at 254 and 398 nm. Peaks with a response exceeding 500 mV were considered the major constituents. For final purification, target compounds were isolated via semipreparative HPLC. The purified compound was dissolved in deuterated DMSO for structural elucidation. NMR spectra (^1^H and ^13^C) were recorded on a Bruker 400 MHz spectrometer, and high-resolution mass spectrometry (HR-ESI-MS) data were obtained using a microTOF-Q mass spectrometer.

## Results

### Morphological and cultural characteristics of strain HMX112^T^

**FIGURE 1:**
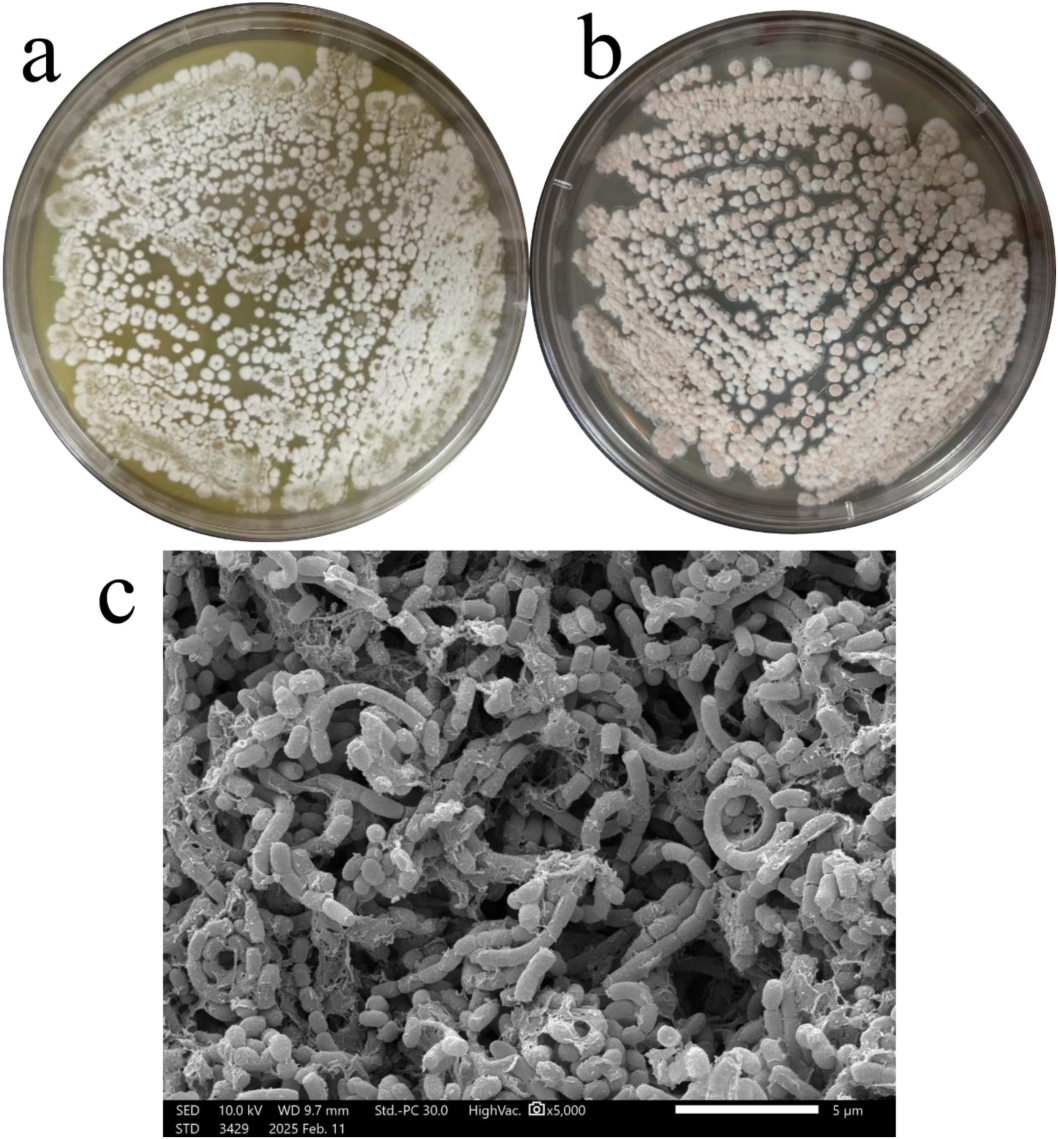
Colony morphology and SEM of strain HMX112. Morphological characteristics of strain HMX112 on Gause No. 1 medium **(a)**, yeast-starch medium **(b)**, and under scanning electron microscopy **(c)**.

Strain HMX112^T^ was cultivated on both Gauze’s No. 1 and yeast-starch agar media at 30°C for 5 days to observe its morphological characteristics. Although growth was faster on the yeast-starch medium, distinct morphological features were evident on each medium. On Gauze’s No. 1 agar, the colony developed yellow substrate mycelia with white aerial mycelia (Figure 1a). In contrast, colonies on yeast-starch agar were pink, and exhibited filiform margins, which are typical of Actinomycetes (Figure 1b). SEM further revealed abundant aerial mycelia bearing spore chains of varying lengths. The spores themselves were ovoid with a smooth, spineless surface, and the spore chains were arranged in curved, ring-like structures (Figure 1c).

### Phylogenetic analysis based on 16s rRNA gene sequences

**FIGURE 2:**
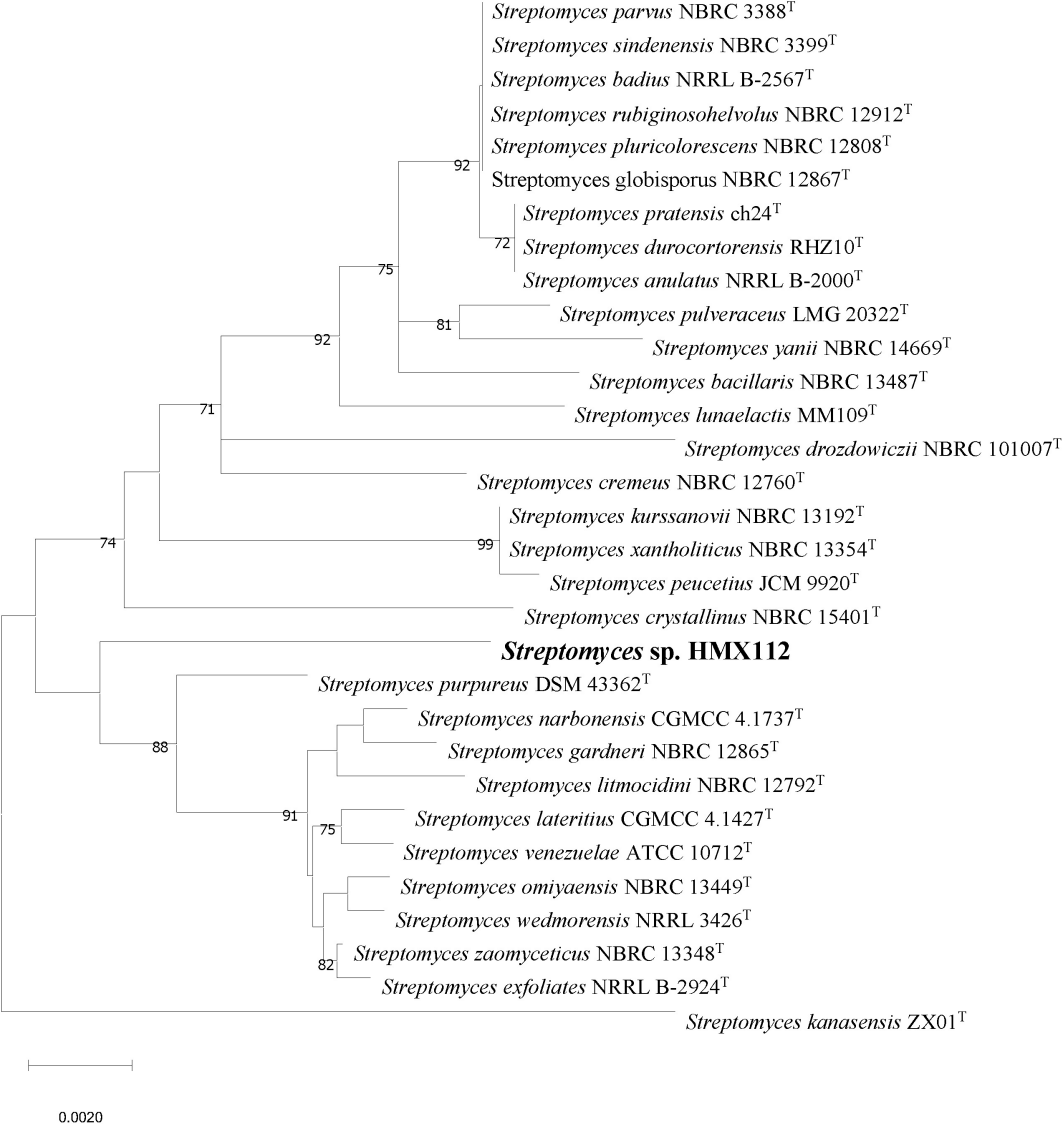
Phylogenetic tree constructed by neighbor-joining method between strain HMX112 and its similar strains.

The nearly complete 16S rRNA gene sequence (1,525 bp) of strain HMX112 was obtained and subjected to analysis using the EzBioCloud database with BLAST. Sequence comparison revealed that HMX112 belongs to the genus *Streptomyces*, showing the highest similarities to *Streptomyces lateritius* CGMCC 4.1427^T^ (98.83%), *Streptomyces narbonensis* CGMCC 4.1737^T^ (98.83%), and *Streptomyces purpureus* DSM 43362^T^ (98.76%). According to the standard criteria for delineating *Streptomyces* species, a 16S rRNA gene sequence similarity below 98.65% suggests a potential novel species. A phylogenetic tree was reconstructed using the neighbor-joining method, incorporating the sequences of 30 strains with similarities greater than 98.2% to HMX112. As shown in Figure 2, while HMX112 clusters with *S. purpureus* DSM 43362^T^, *S. narbonensis* CGMCC 4.1737^T^, and *S. lateritius* CGMCC 4.1427^T^, it forms a distinct monophyletic branch. This clear separation indicates significant sequence divergence from other closely related species, sufficiently supporting the status of HMX112 as a novel species. The 16S rRNA gene sequence of strain HMX112 has been deposited in the GenBank database under the accession number PQ182579.

### Analysis of physiological and biochemical characteristics

Physiological characterization revealed that strain HMX112 grew at temperatures ranging from 25 to 37°C (optimum 25–30°C), a pH of 5–11 (optimum 7–8), and tolerated NaCl concentrations up to 5%. Substrate utilization profiling using Biolog GEN III microplates indicated that the strain could utilize a variety of carbon sources, including sugars (e.g., D-maltose, D-trehalose, D-cellobiose, sucrose, D-salicin, D-galactose, D-fructose, D-mannose, α-D-glucose, glycerol), amino sugars (e.g., N-acetyl-β-D-glucosamine), and organic acids (e.g., citric acid, acetoacetic acid, propionic acid). For nitrogen sources, it utilized amino acids such as glycyl-L-proline, L-alanine, L-aspartic acid, L-glutamic acid, and L-serine. The strain tested positive for gelatin hydrolysis. Furthermore, it showed positive reactions for nalidixic acid and lithium chloride, and exhibited tolerance to aztreonam and sodium bromate. Notably, strain HMX112 exhibits antibacterial activity against the tested microorganisms. A detailed comparative summary of the physiological and biochemical characteristics of strain HMX112 and its closely related type strains is provided in Table 1.

**TABLE 1:** Differentiation characteristics of strain HMX112 and type strains of phylogenetically closely related species of the genus *Streptomyces*.

Characteristic	Strain 1	Strain 2	Strain 3	Strain 4
Optimal growth temperature (°C)	25–30	30	25	25–30
pH tolerance range	5–11	6–12	5–12	6–12
NaCl tolerance range (%)	0–5	0–5	0–5	0–7
D-maltose	+	+	+	+
D-trehalose	+	+	(+)	+
D-cellobiose	+	(+)	+	+
Sucrose	(+)	+	+	+
D-salicin	+	+	+	+
D-galactose	+	+	+	+
D-fructose	+	(+)	+	+
D-mannose	+	+	+	+
α-D-glucose	+	(+)	+	+
Glycerol	+	+	+	+
Pectin	**-**	(+)	(+)	(+)
D-galacturonic acid	(+)	+	(+)	(+)
L-galacturonic acid lactone	(+)	+	(+)	+
N-acetyl-β-D-glucosamine	+	(+)	+	+
N-acetylneuraminic acid	(+)	+	(+)	(+)
N-acetyl-D-galactosamine	(+)	+	(+)	(+)
Citric acid	+	+	(+)	(+)
D-lactic acid methyl ester	(+)	(+)	(+)	(+)
Acetoacetic acid	+	+	(+)	(+)
Propionic acid	+	+	+	+
α-ketoglutaric acid	(+)	+	+	(+)
D-malic acid	(+)	+	(+)	**-**
Aminoacetyl-L-proline	+	+	+	+
L-alanine	+	+	(+)	+
L-aspartic acid	+	+	+	(+)
L-glutamic acid	+	+	+	+
L-serine	+	+	(+)	(+)
D-serine	**-**	**-**	(+)	**-**
Gelatin	+	(+)	+	+
Tween 40	+	**-**	+	+
Acetochlor	**-**	(+)	**-**	(+)
Rifamycin	**-**	+	**-**	(+)
Naphthoquinone acid	+	+	+	+
Lithium chloride	+	+	(+)	+
Amikacin	(+)	+	+	+
Sodium bromate	(+)	(+)	**-**	(+)

Strain 1, Strain HMX112; Strain 2, *Streptomyces lateritius* CGMCC 4.1427^T^; Strain 3, *Streptomyces narbonensis* DSM 40016^T^; Strain 4, *Streptomyces purpureus* DSM 43362^T^. +, Positive; -, Negative; (+), Borderline.

**FIGURE 3:**
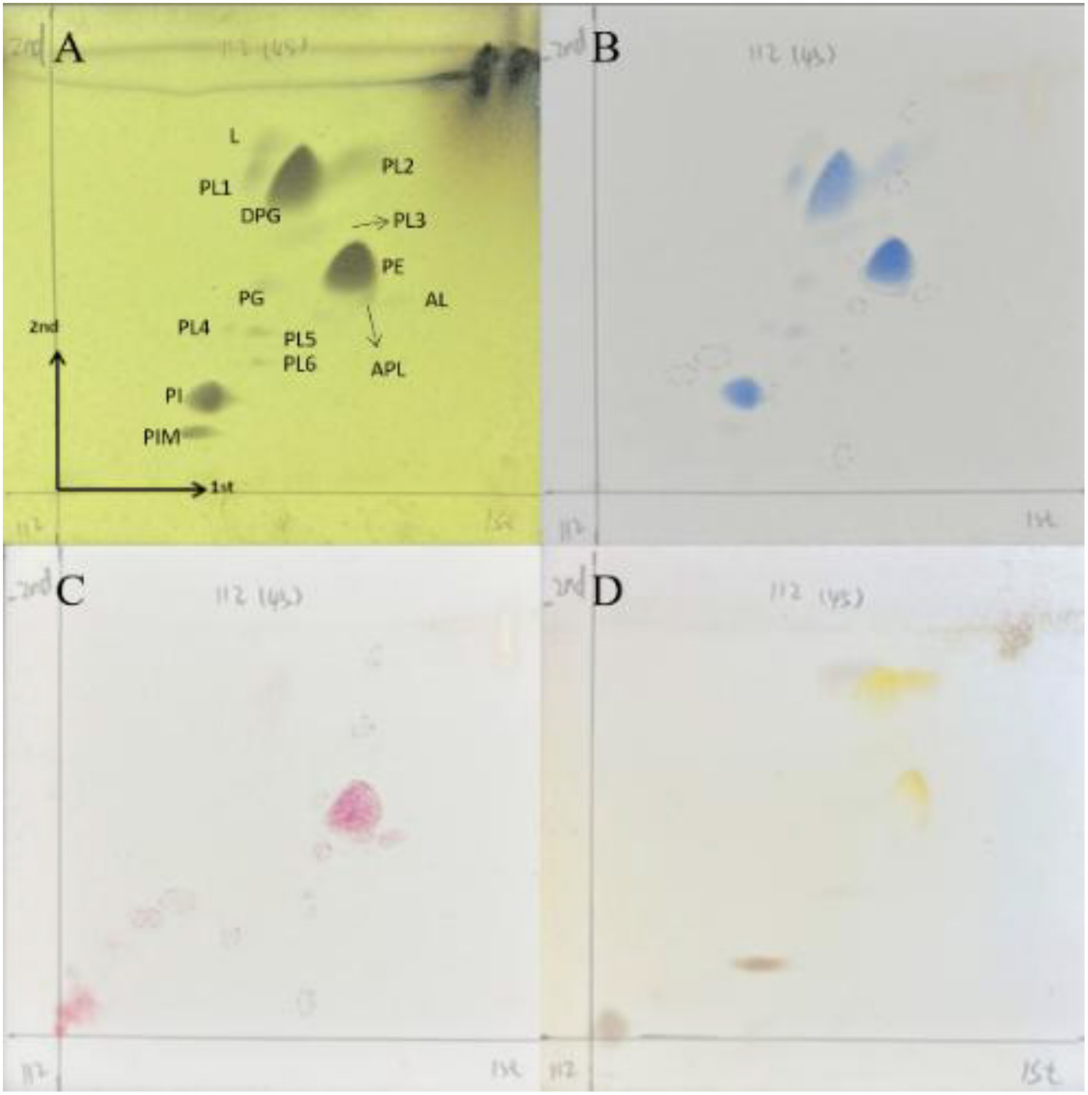
Polar lipid profile of strain HMX112. Chemical detection of polar lipids on the TLC plate was performed using the following specific spray reagents: **(A)** phosphomolybdate for total lipids; **(B)** molybdenum blue for phospholipids; **(C)** ninhydrin for aminolipids; **(D)** α-naphthol (1-naphthol) for glycolipids.

Chemotaxonomic analysis of strain HMX112 confirmed its classification within the genus *Streptomyces*. The cell wall was found to contain LL-diaminopimelic acid (LL-DAP), and the whole-cell hydrolysates included ribose and glucose. The predominant menaquinones were identified as MK-9(H8) (65.94%), MK-10(H4) (23.95%), and MK-9(H4) (10.11%). The polar lipid profile consisted of diphosphatidylglycerol (DPG), phosphatidylglycerol (PG), phosphatidylinositol (PI), phosphotidylethanolamine (PE), phosphatidylinositol mannosides (PIM), and several unidentified lipids (L, AL, APL, PL) (Figure 3). The major cellular fatty acids were iso-C16:0 (27.94%), anteiso-C15:0 (14.15%), and anteiso-C17:0 (9.68%). A detailed comparative fatty acid composition between strain HMX112 and its closely related type strains is provided in Table 2.

**TABLE 2:** Cellular fatty acid composition of strain HMX112 and its closest type strains.

Fatty acids	Strain 1	Strain 2	Strain 3	Strain 4
C_14:0_	–	–	1.43	0.40
C_15:0_	–	1.03	0.96	0.48
C_16:0_	3.94	4.06	5.36	5.20
C_18:0_	3.46	0.37	0.28	–
Iso-C_12:0_	–	–	1.68	–
Iso-C_13:0_	–	–	–	0.14
Iso-C_14:0_	3.46	2.06	3.05	6.22
Iso-C_15:0_	6.93	10.20	6.67	7.93
Anteiso-C_13:0_	–	–	0.19	0.13
Anteiso-C_15:0_	14.15	23.00	19.76	21.58
Anteiso-C_16:0_	–	–	0.32	0.59
Iso-C_16:0_	27.94	16.57	20.72	21.44
Iso-C_16:1_ H	7.44	0.63	2.58	2.04
Sum in feature 3	2.37	0.56	1.51	0.16
Sum in feature 5	–	–	–	0.24
Sum in feature 8	–	0.36	–	0.17
Sum in feature 9	4.24	2.82	3.02	1.33
Iso-C_17:0_	2.17	10.06	2.39	2.91
Anteiso-C_17:0_	9.68	15.46	8.19	8.81
Cyclo-C_17:0_	3.51	1.97	2.73	3.38
Cyclo-C_19:0_ ω8c	–	–	0.40	–
Anteiso-C_17:1_ ω9c	6.10	3.50	4.96	6.99
Iso-C_18:1_ H	1.49	–	1.39	–
Iso-C_18:0_	3.22	2.34	1.64	5.76
C_17:0_ 2OH	–	–	–	0.20
C_17:1_ ω8c	0.89	–	0.25	–
C_18:1_ ω9c	1.63	–	–	–
Summed feature 3	2.37	0.56	1.51	0.16
Summed feature 5	–	–	–	0.24
Summed feature 8	–	0.36	–	0.17
Summed feature 9	4.24	2.82	3.02	1.33

Strain 1, StrainHMX112; Strain 2, *Streptomyces lateritius* CGMCC 4.1427^T^; Strain 3, *Streptomyces narbonensis* CGMCC 4.1737^T^; Strain 4, *Streptomyces purpureus* DSM 43362^T^. Fatty acids are indicated using standard abbreviations; iso- and anteiso- denote branched chains, 3OH indicates hydroxylation, and double-bond positions denote unsaturation. “Sum in feature X” represents the total percentage of fatty acids included in feature X as defined by the MIDI system (v6.0). “Summed feature 7, 8, 9” correspond to co-eluting fatty acids that cannot be re-solved individually by GC. “–,” not detected. Values in the table represent percentages.

### Whole-genome sequencing of strain HMX112^T^

**FIGURE 4:**
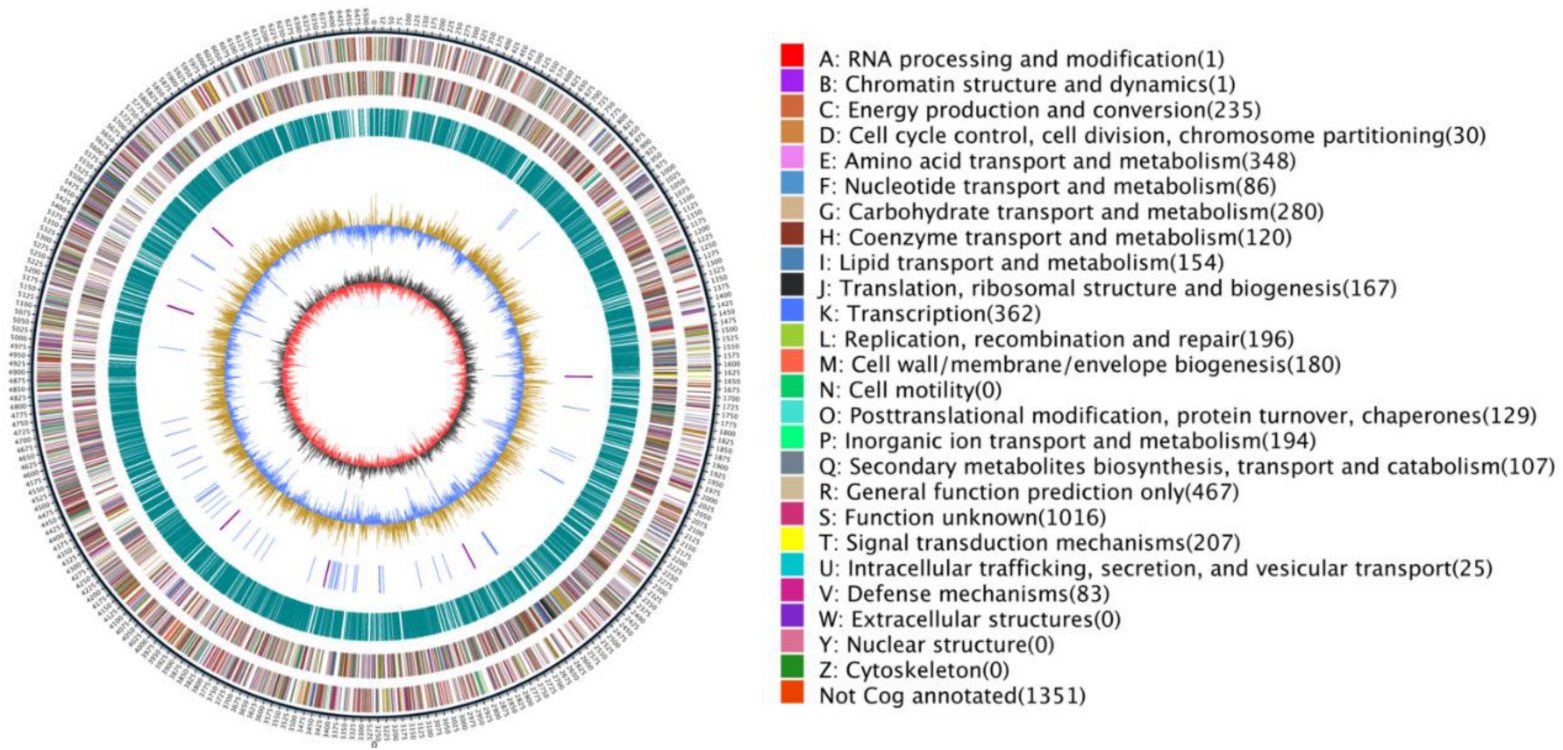
Circos plot of the genome of strain HMX112^T^.

The complete genome of strain HMX112^T^ was sequenced and assembled, revealing a circular chromosome of 6,523,537 bp with a GC content of 72.93% (Figure 4). The genome was predicted to contain 5,739 protein-coding genes, 18 rRNA, and 67 tRNA genes. To unequivocally determine its taxonomic status, the ANI and (dDDH) values between HMX112^T^ and its three closest phylogenetic relatives were calculated. As shown in Table 3, both the ANI and dDDH values were significantly below the established thresholds for species delineation (95 and 70%, respectively). Based on a comprehensive polyphasic taxonomic approach that integrates distinct phenotypic, physiological, chemotaxonomic, and genomic characteristics, as well as its unique ability to produce a fluorescent yellow antibacterial compound, strain HMX112^T^ is proposed to represent a novel species of the genus *Streptomyces*. We propose the name *Streptomyces flavimicrosus* sp. nov. for this organism. The whole-genome sequence has been deposited in the NCBI GenBank database under the BioProject accession number PRJNA1206132.

**TABLE 3:** 16S rRNA identity, ANI, and DDH genomic comparisons between strains HMX112 and their closest phylogenetic neighbors.

Strain 1	Strain 2	16S rRNA identity	ANI	DDH
HMX112	*S. lateritius* LMG 19372^T^	98.83	84.0336	28
	*S. narbonensis* NBRC 12801^T^	98.83	83.2872	24.8
	*S. purpureus* NBRC 13927^T^	98.76	84.4736	28.6

### Comparative genomic analysis of four *Streptomyces* species

**FIGURE 5:**
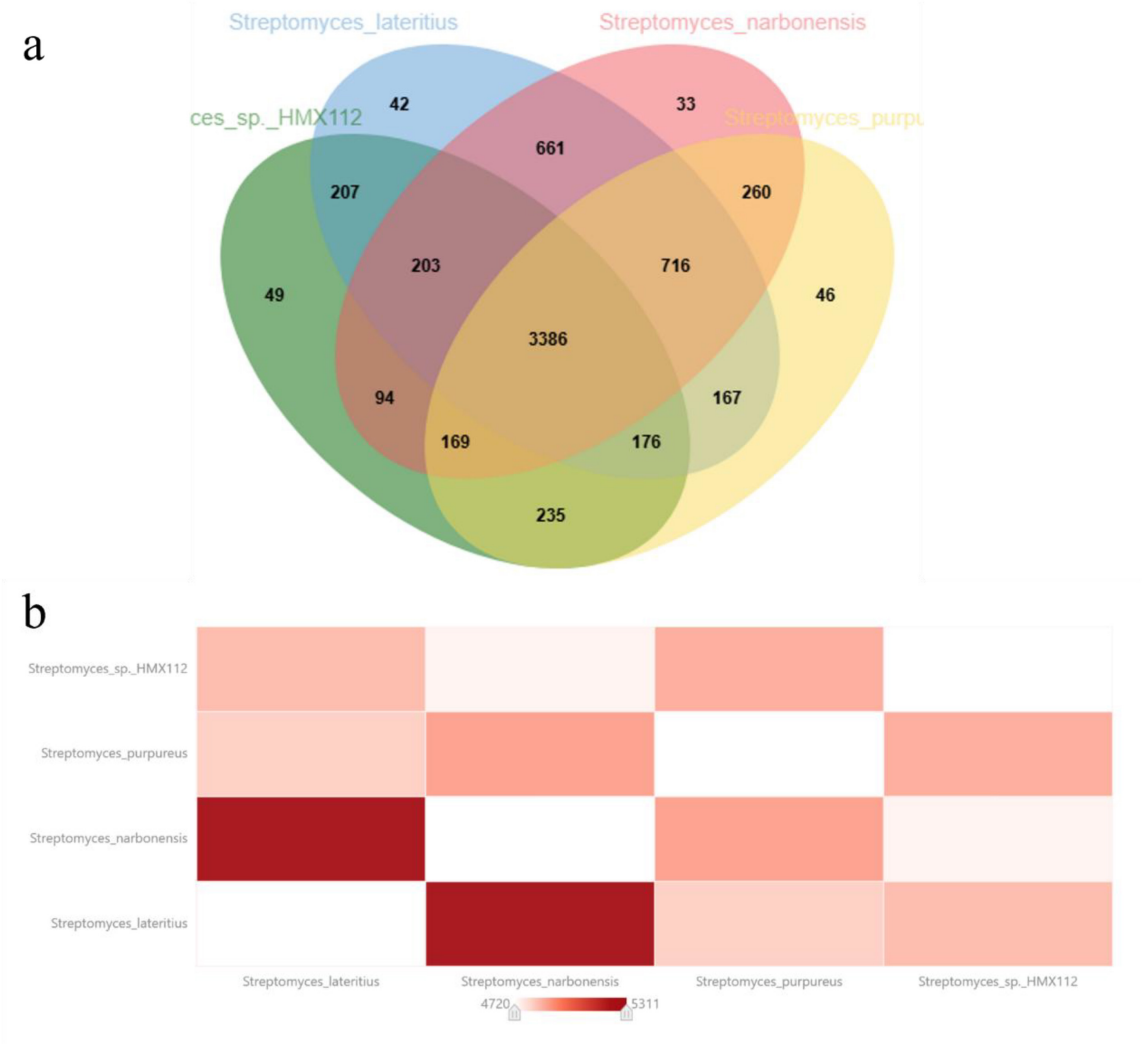
Pan-genomic analysis and genomic covariance analysis plots of four *Streptomyces* species. **(a)** Venn diagram of the four *Streptomyces* species, **(b)** clustering heatmap of the four *Streptomyces* species.

A comparative genomic analysis based on homologous protein-coding genes was conducted for *Streptomyces flavimicrosus* sp. nov. HMX112^T^ and its three closely related strains. The Venn diagram (Figure 5a) reveals a core genome of 3,386 orthologous gene clusters shared among all four strains, representing 60.9–74.9% of each strain’s total gene content, which likely underlies their essential biological functions. The non-overlapping sections represent strain-specific gene sets. *S. flavimicrosus* HMX112^T^ possesses 49 (1.08%) unique genes, compared to 42 (0.76%) in *S. lateritius* CGMCC 4.1427^T^, 33 (0.6%) in *S. narbonensis* DSM 40016T, and 46 (0.89%) in *S. purpureus* DSM 43362T. Although HMX112^T^ has a slightly smaller total genome size, it harbors a comparatively higher proportion of unique genes, suggesting it may encode distinct metabolic capabilities. Furthermore, a genome-wide synteny analysis (Figure 5b) indicated a higher degree of collinearity between HMX112^T^ and *S. purpureus* DSM 43362^T^, while revealing genomic rearrangements or indels relative to *S. narbonensis* DSM 40016^T^. In contrast, *S. lateritius* CGMCC 4.1427^T^ and *S. narbonensis* DSM 40016^T^ exhibited the highest level of synteny, indicating a closer genomic relationship between them.

### Genomic features for adaptation to extreme environments of strain HMX112^T^

**FIGURE 6:**
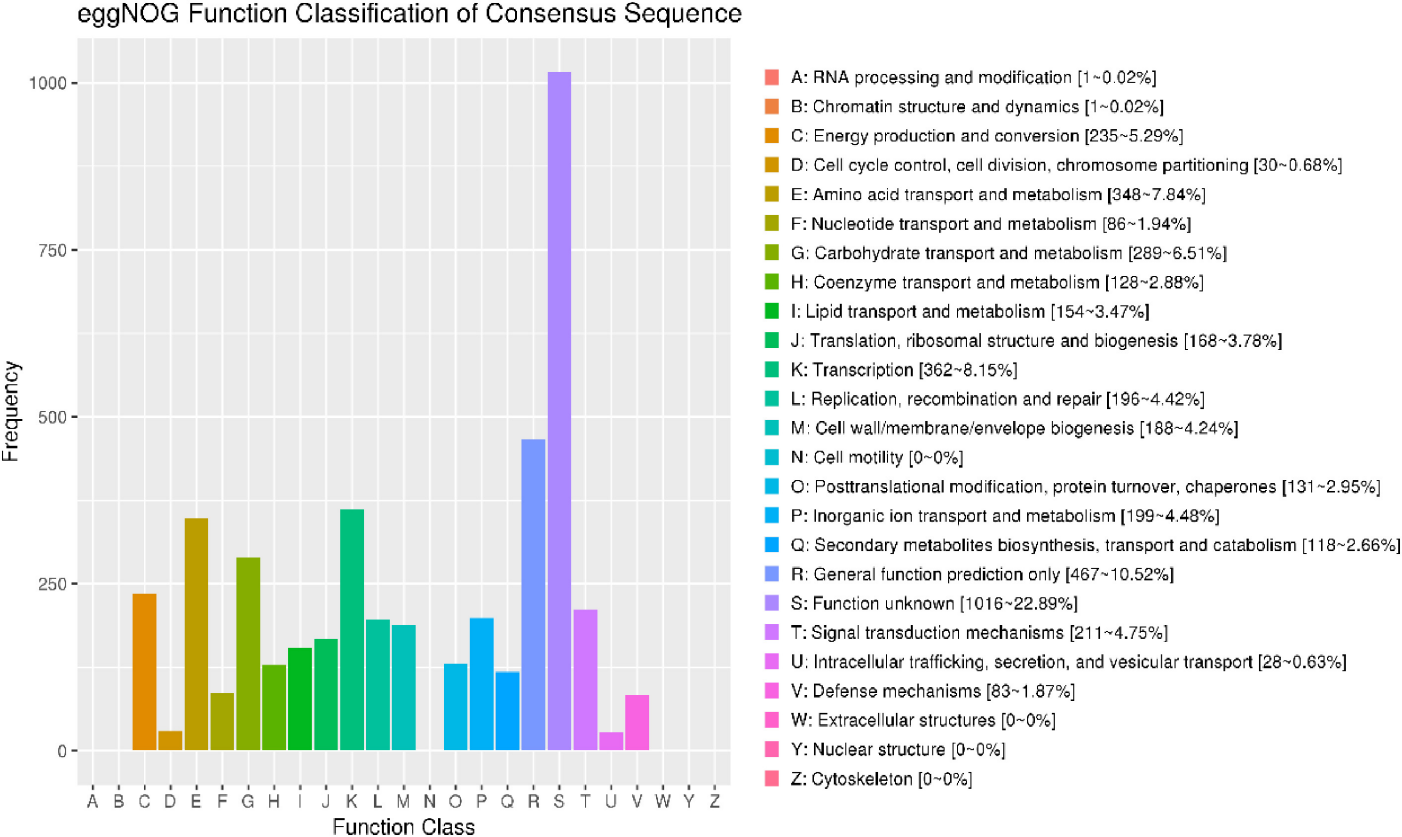
EggNOG functional classification chart for strain HMX112^T^.

Functional annotation of the predicted protein-coding genes of strain HMX112T was performed using the eggNOG database, revealing a diverse functional profile (Figure 6). A high proportion of genes were assigned to transcription (Category K, 8.15%) and amino acid transport and metabolism (Category E, 7.84%), indicating robust capabilities in gene expression regulation and nitrogen metabolism, consistent with its physiological capacity to utilize various amino acids such as L-aspartate and L-glutamate. Significant enrichment was observed in carbohydrate transport and metabolism (Category G, 6.51%), reflecting the strain’s ability to utilize a broad spectrum of carbon sources, including D-maltose and D-trehalose. In the context of environmental adaptation and stress response, a notable abundance of genes related to signal transduction mechanisms (Category T, 4.75%) and inorganic ion transport and metabolism (Category P, 4.48%) suggests molecular mechanisms underlying its adaptation to saline-alkaline conditions (0–5% NaCl tolerance) and oxidative stress. Genes associated with defense mechanisms (Category V, 1.87%) are likely involved in its tolerance to nalidixic acid and lithium chloride. With respect to secondary metabolic potential, genes involved in the biosynthesis, transport, and catabolism of secondary metabolites (Category Q, 2.66%), including non-ribosomal peptide synthetases (NRPS) and polyketide synthases (PKS), indicate a capacity for producing antibacterial or antifungal compounds. Lipid metabolism genes (Category I, 3.47%) may contribute to membrane stability under osmotic stress in desert environments. Notably, the largest proportion of genes (Category S, 22.89%) were annotated as unknown, suggesting the presence of uncharacterized genetic determinants that may contribute to unique ecological adaptations or novel secondary metabolic pathways. Furthermore, the complete absence of genes related to cell motility (Category N), extracellular structures (Category W), nuclear structure (Category Y), and cytoskeleton (Category Z) aligns with typical *Streptomyces* characteristics, including the lack of flagellar motility and eukaryotic-like cytoskeletal structures.

### The prediction for secondary metabolite production in the genome of strain HMX112^T^

To gain deeper insights into the antibacterial functional characteristics of strain HMX112^T^, its protein sequences were analyzed using the Comprehensive Antibiotic Research Database (CARD). This analysis identified four antibiotic resistance genes and their associated resistance mechanisms (Table 4). MexW is a RND-type membrane fusion protein component of the MexVW-OprM efflux complex, which confers resistance to multiple antibiotics, including macrolides, acridine dyes, tetracyclines, fluoroquinolones, and phenolics, through an antibiotic efflux mechanism. IleS, a naturally occurring resistance gene in bifidobacteria encoding isoleucyl-tRNA synthetase, confers resistance to mupirocin via target alteration. IMP-51, a variant of metallo-β-lactamase (MBL) belonging to the IMP family of carbapenemases, inactivates multiple β-lactam antibiotics, including penicillins, cephalosporins, cephamycins, and carbapenems, through antibiotic inactivation. MupB is an alternate isoleucyl-tRNA synthetase that also confers mupirocin resistance via target alteration. Mupirocin is an antibiotic used to treat skin infections, particularly those caused by MRSA. The presence of ileS and mupB genes, which encode isoleucyl-tRNA synthetase or its variant, can render a strain insensitive to mupirocin, enabling survival and growth in its presence. The identification of both ileS and mupB in strain HMX112^T^ suggests that this strain may either produce mupirocin-like compounds or possess intrinsic mechanisms to survive in the presence of this antibiotic. Consequently, HMX112^T^ represents a promising candidate for the discovery of novel anti-MRSA antibiotics or unique bioactive metabolites. Furthermore, this novel *Streptomyces* species could serve as a new chassis organism, providing fresh perspectives for studying antibiotic resistance mechanisms.

**TABLE 4:** The resistance gene of HMX112^T^.

Gene ID	Name	Resistance
GE003246	*MexW*	Macrolide, acridine dyes, tetracycline, fluoroquinolone, phenolic antibiotics
GE001188	Bifidobacterium *ileS* conferring resistance to mupirocin	Mupirocin antibiotic
GE004709	*IMP-51*	Pipramic acids, peramic acids, carbapenems, cephamycins, cephamycins
GE005721	Staphylococcus *mupB* conferring resistance to mupirocin	Mupirocin antibiotic

Genome mining of *Streptomyces flavimicrosus* sp. nov. HMX112^T^ using antiSMASH predicted 31 BGCs, encompassing diverse types such as NRPS, PKS, terpenes, and ribosomally synthesized and post translationally modified peptides (RiPPs) (Table 5). Notably, 19 of these BGCs exhibited low similarity to known clusters, indicating a high potential for the production of novel chemical scaffolds. Several high similarity BGCs (e.g., BGC1, BGC21, BGC24, and BGC28) showed strong homology to known pathways for antimicrobial peptides, siderophores, and osmoprotectants, underscoring the metabolic versatility of HMX112^T^. Importantly, NRPS and PKS clusters collectively accounted for nearly 40% of all predicted BGCs, aligning with the major structural types of anti-MRSA compounds derived from *Streptomyces*. This suggests significant potential of HMX112^T^ in combating multidrug-resistant pathogens like MRSA. Furthermore, low similarity clusters (e.g., BGC4, BGC8, and BGC10) may encode previously uncharacterized metabolic pathways, providing promising targets for the discovery of new natural products. These findings collectively highlight the value of HMX112^T^ as a promising source for novel antibiotic discovery, particularly in the screening for compounds active against multidrug-resistant bacteria.

**TABLE 5:** Potential BGCs for secondary metabolites in *Streptomyces flavimicrosus* sp. nov. HMX112^T^.

BGC	Type	Similar known gene cluster	Similarity
1	NRPS, indole, azole-containing-RiPP	Streptamidine	High
2	Melanin	Melanin	Low
3	Terpene	Isorenieratene	High
4	T1PKS, NRPS	–	–
5	T3PKS	–	–
6	NRPS	–	–
7	Terpene	Hopene	Medium
8	RiPP-like, redox-cofactor	–	–
9	Ectoine	–	–
10	RiPP-like	14-hydroxyisochainin	Low
11	Lanthipeptide-class-i	–	–
12	Hydrogen-cyanide	Aborycin	Low
13	NRPS	Cyclofaulknamycin	Low
14	NI-siderophore	Kinamycin	Low
15	Other	–	–
16	Terpene-precurso	–	–
17	Melain	–	–
18	Aminopolycarboxylic-acid	Ethylenediaminesuccinic acid hydroxyarginine	High
19	NI-siderophore	–	–
20	T2PKS, butyrolactone	Griseusin	Medium
21	RRE-containing, azole-containing-RiPP	Berninamycin K/berninamycin J/berninamycin A/berninamycin B	Medium
22	NI-siderophore	Desferrioxamin B/desferrioxamine E	High
23	Butyrolactone	–	–
24	NRPS	Pyrroloformamide A/pyrroloformamide B/pyrroloformamide D/pyrroloformamide C	Low
25	T3PKS, ectoine	Ectoine	High
26	NRPS-like	Deoxyhangtaimycin	Low
27	NRPS-like, betalactone	–	–
28	T3PKS, lanthipeptide-class-iii	SapB	High
29	Terpene	Geosmin	High
30	NRPS-like, NRPS	Antipain	High
31	RRE-containing, T3PKS	Flaviolin	Medium

### Isolation, identification, and antimicrobial activity of active natural products from strain HMX112^T^

**FIGURE 7:**
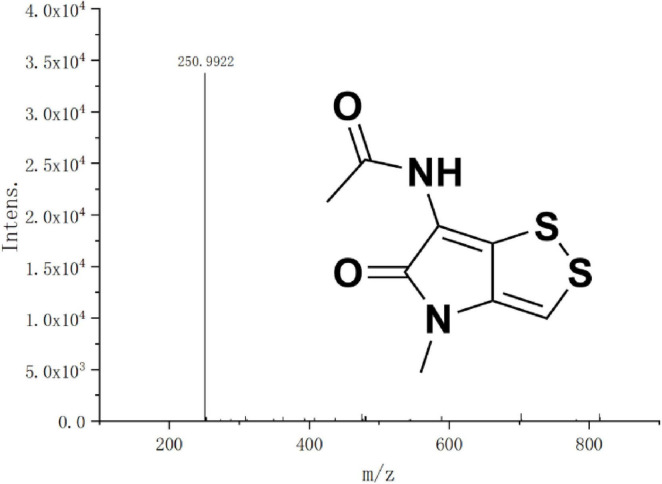
Mass-to-core ratio [(M+Na)^+^] and chemical structure formula of the compound.

The crude ethyl acetate extract from the 7-day fermentation broth of strain HMX112T was fractionated by normal-phase column chromatography. Further purification was achieved via preparative thin-layer chromatography, yielding the target bright yellow band as the pure compound. The compound appeared as a bright yellow flocculent solid under natural light, and exhibited poor solubility in common organic solvents, being slightly soluble in chloroform and acetone but more soluble in DMSO (6.25 mg/mL), while insoluble in water. As shown in Figure 7, high-resolution mass spectrometry (HRMS) indicated an [M+Na]^+^ ion at m/z 250.9922, and analysis of ^13^C NMR data supported the molecular formula C_8_H_8_N_2_O_2_S_2_, with six degrees of unsaturation. The ^13^C NMR spectrum revealed eight carbon signals: two ester carbonyl carbons (δC 166.1 and 168.8), four aromatic carbons (δC 111.0, 114.8, 132.4, and 136.0), and two methyl carbons (δC 22.4 and 27.5). Key features in the ^1^H NMR spectrum included a doublet at δH 7.34, assigned to the aromatic proton (H-3) of the thiolopyrrolone ring, likely split by weak coupling to a sulfur atom; a singlet at δH 2.02, corresponding to the methyl group of an acetoxy moiety; a singlet at δH 3.25 for the methoxy methyl group, with its downfield carbon shift (δC 27.5) suggesting the influence of an adjacent thioester group; and a broad singlet at δH 9.98, characteristic of an amide proton (–NH). Isotopic peak analysis of the mass spectrum showed an M+2/M ratio of approximately 10%, consistent with the theoretical abundance for two sulfur atoms (∼9%) and ruling out the presence of chlorine. The NMR data further supported a fused thiolopyrrolone ring system, with a sulfur atom bridging C-3a (δC 136.0) and C-6a (δC 132.4) to form a rigid planar structure. By comparing these data with literature values (Song et al., 2022) and known compound characteristics, the isolated compound was conclusively identified as thiolutin. A detailed comparative summary is provided in Table 6.

**TABLE 6:** Comparison of ^1^H-NMR and ^13^C-NMR data of compound and compound thiolutin.

The compound		The known compound thiolutin	
δ _H_	*δ _*C*_*	δ _H_	*δ _*C*_*
7.34 (d, 1.0, 1H)	111.0	7.34 s	111.0
	136.0		136.0
	166.1		166.1
	114.8		114.1
	132.4		132.4
	168.8		168.8
2.02 (s, 3H)	22.4	2.02 s	22.4
3.25 (s, 3H)	27.5	3.23 s	27.5
9.98 s		9.99 s	

This study systematically evaluated the antimicrobial activity of thiolutin, demonstrating its broad-spectrum potential (Table 7). In assays against common pathogenic bacteria, thiolutin exhibited significant inhibitory effects against all seven tested strains of both Gram-positive and Gram-negative bacteria. Notably, the zones of inhibition against *Staphylococcus pasteuri* and *Staphylococcus epidermidis* (27 mm and 20 mm, respectively) exceeded those produced by the positive control kanamycin, indicating superior efficacy against certain Gram-positive bacteria compared to this conventional antibiotic. In evaluations against multidrug-resistant bacteria, thiolutin showed pronounced activity against MRSA strains (including ATCC29213, N315, and two clinical isolates), with MIC values ranging from 8 to 16 μg/mL, suggesting promising potential against clinical isolates. Furthermore, at a concentration of 50 ppm, thiolutin achieved 100% inhibition against several plant pathogenic fungi, including *Fusarium graminearum* and *Rhizoctonia solani*, while demonstrating varying degrees of suppression against other common fungal pathogens. Its antimicrobial mechanism is thought to involve inhibition of bacterial RNA polymerase, disruption of transcription, and potential interference with fungal metabolic pathways or cell wall synthesis, collectively contributing to its broad-spectrum action. In conclusion, thiolutin shows considerable promise for application against pathogenic bacteria, including drug-resistant strains, as well as plant pathogenic fungi.

**TABLE 7:** Thiolutin bacteriostatic activity test results.

Antibacterial zone diameter (mm)		*Klebsiella pneumoniae*		*Escherichia coli*			*Bacillus thuringiensis*		*Pseudomonas aeruginosa*		*Staphylococcus aureus*			*Staphylococcus pasteuri*		*Staphylococcus epidermidis*
Thiolutin (1.75 mg/mL)		14		17			14.5		8		17.5			20		27
Kanamycin (100 mg/mL)		27		30			32		19		20.5			10		23
MIC (μg/mL)		ATCC29213				N315			Clinical strain 1					Clinical strain 2		
Thiolutin		16				16			8					16		
Vancomycin		1				1			2					2		
Antibacterial rate %	1	2	3		4		5	6		7		8	9		10	11
Thiolutin (50μg/mL)	100	81.8	27.10		75.63		100	72.8		76.2		82.6	100		52.3	100

1, *F. graminearum*; 2, *F. oxysporum*; 3, *F. solani*; 4, *R. solani*; 5, *S. sclerotiorum*; 6, *A. tenuissima*; 7, *A. solani*; 8, *B. cinerea*; 9, *P. capsici*; 10, *C. fructicola*; 11, *Valsa mali.*

### Analysis of the biosynthetic mechanism of thiolutin

Genomic analysis of *Streptomyces flavimicrosus* sp. nov. HMX112^T^ revealed that its cluster 24 encodes a predicted product containing a pyrrolone ring structure, showing high similarity to the core scaffold of thiolutin-like compounds. To further assess the phylogenetic position of this gene cluster, we performed BLASTX analysis on all genes (see Supplementary File 1 for details). Results indicate that the core biosynthetic genes within this cluster (e.g., the nonribosomal peptide synthetase gene ctg1_4204) and the vast majority of accessory genes share the highest homology (over 98% sequence identity, E-value ≈ 0) with hypothetical or functionally annotated proteins from *Streptomyces* sp. NPDC050856 (consistency > 98%, E-value ≈ 0). Notably, a few genes (e.g., ctg1_4213, ctg1_4214) showed optimal matches to other *Streptomyces* species, suggesting that this gene cluster may have undergone complex recombination events during evolution.

**FIGURE 8:**
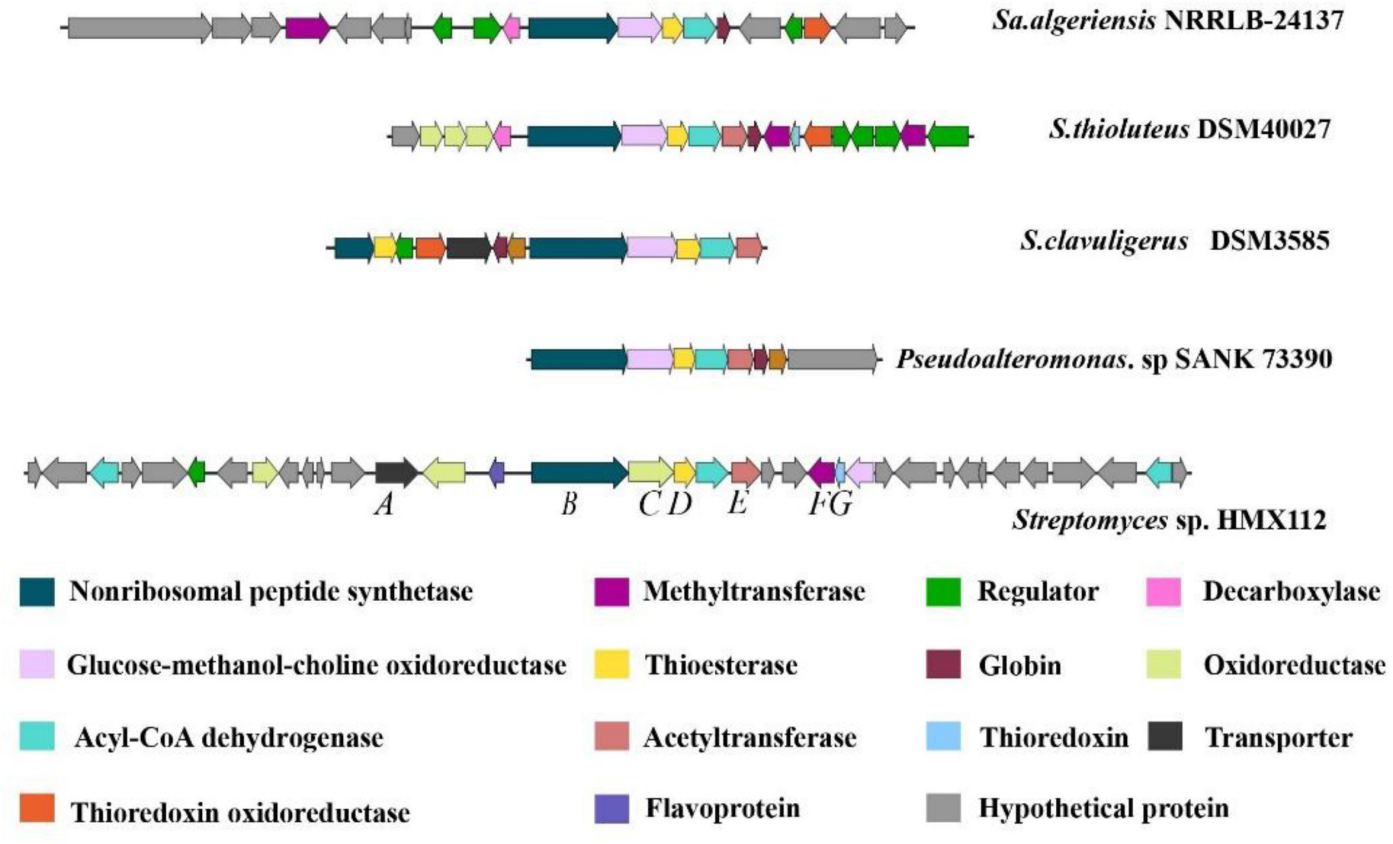
Comparative analysis of the thiolutin biosynthetic gene cluster (cluster *thi*) in *Streptomyces flavimicrosus* HMX112^T^ with known dithiolopyrrolone BGCs.

To further investigate the biosynthetic pathway of thiolutin in HMX112^T^, we compared this cluster with four known gene clusters responsible for the production of thiolutin or its analogs: the holomycin cluster from *Streptomyces clavuligerus* DSM40027, the thiolutin cluster from *Saccharothrix algeriensis* NRRLB-24137, the aureothricin cluster from *Streptomyces thioluteus* DSM40027, and the thiomarinol cluster from *Pseudoalteromonas* sp. SANK 73390. Comparative analysis revealed a highly conserved organization of core biosynthetic genes across all four clusters (Figure 8). All clusters shared genes encoding a nonribosomal peptide synthetase (NRPS), a thioesterase, and an acyl-CoA dehydrogenase. Based on this high degree of conservation, cluster 24 was preliminarily identified as the thiolutin biosynthetic gene cluster and designated as cluster *thi*.

The NRPS (ThiB) serves as the central catalytic unit responsible for initiating biosynthesis. Its adenylation (A) domain specifically recognizes and activates two cysteine substrates (Conti et al., 1997, May et al., 2002, Stachelhaus et al., 1999). The peptidyl carrier protein (PCP) transports the activated intermediates (Weber et al., 2000), and the condensation (C) domain catalyzes peptide bond formation and cyclization to form the core dithiolopyrrolone ring scaffold (Keating et al., 2002). Working in close concert with the NRPS, the thioesterase (ThiD) hydrolyzes the mature cyclized intermediate from the NRPS, releasing it from the enzyme complex. An acetyltransferase (ThiE) is a key tailoring enzyme that determines structural diversity; it likely utilizes different acyl-CoA donors to acylate the N7 position of the cyclic scaffold, leading to the production of thiolutin or structural analogs. The formation of the disulfide bridge within the dithiolopyrrolone ring is facilitated by oxidoreductases within the cluster, such as a flavoprotein and other redox enzymes, which help maintain the requisite redox environment.

**FIGURE 9:**
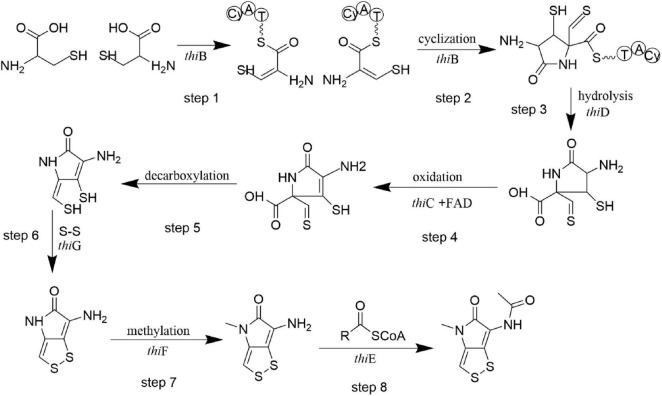
The biosynthetic pathway of thiolutin in *Streptomyces flavimicrosus* HMX112^T^.

Based on our analysis, we propose the biosynthetic pathway for thiolutin in HMX112^T^ (Figure 9). The process is initiated by the NRPS (ThiB), which activates and loads two cysteine molecules. Its condensation domain then catalyzes peptide bond formation coupled with cyclization, generating a dipeptide-derived heterocyclic intermediate that remains tethered to the NRPS via a thioester bond. The thioesterase (ThiD) subsequently hydrolyzes this bond, releasing the cyclic scaffold. This is followed by the action of an oxidoreductase (ThiC), potentially assisted by other auxiliary redox enzymes, leading to the formation of the preliminary dithiolopyrrolone structure. The scaffold subsequently undergoes decarboxylation, a step potentially catalyzed by an unannotated decarboxylase within the cluster or a promiscuous enzyme with dual functionality. The decarboxylated, reduced intermediate (holothin) is then oxidized, likely involving the thioredoxin-like protein (ThiG), to form the intramolecular disulfide bond. A methyltransferase (ThiF) subsequently introduces a methyl group at the N^4^ position, yielding a methylated derivative of holothin. Finally, an acyltransferase (ThiE), utilizing propionyl-CoA as a substrate, catalyzes N^7^ acylation to produce the final product, thiolutin, which is subsequently exported from the cell by the transporter (ThiA).

## Discussion

The present study successfully identified *Streptomyces flavimicrosus* sp. nov. HMX112^T^ from the extreme desert environment of the Turpan-Hami Basin. Its status as a novel species was confirmed based on 16S rRNA gene phylogeny, genomic distances (ANI/dDDH values significantly below the 95 and 70% species threshold, respectively), and distinct physiological and chemotaxonomic profiles. This finding reinforces the notion that underexplored ecological niches, such as arid deserts, remain valuable reservoirs for discovering novel microbial taxa.

The adaptation strategies of strain HMX112^T^ to its harsh native habitat appear to be genomically encoded. Its tolerance to environmental stressors (e.g., salinity, oxidative stress) correlates with a rich repertoire of stress-response genes identified in its genome. Several genomic features may underpin this extremophilic lifestyle: a high GC content (72.93%), potentially linked to DNA stability under hot, dry conditions (Galtier et al., 1999); the production of a bright yellow pigment and the presence of a geosmin biosynthetic gene cluster (BGC28, 100% similarity), which may mitigate oxidative damage by scavenging reactive oxygen species or absorbing UV radiation (Gerber and Lechevalier, 1965); and an ectoine synthesis gene cluster (BGC24, 100% similarity) likely involved in osmoregulation (Ali et al., 2022). Furthermore, the substantial proportion of genes with unknown functions (22.89%) offers intriguing targets for elucidating unique adaptive mechanisms and novel metabolic pathways in this desert isolate.

The antibiotic thiolutin, isolated from strain HMX112^T^, demonstrated potent and broad-spectrum antimicrobial activity. Its efficacy against Gram-positive bacteria, including *Staphylococcus pasteuri* and *Staphylococcus epidermidis*, surpassed that of the conventional antibiotic kanamycin. Thiolutin also showed promising activity against multidrug-resistant MRSA strains, with MIC values of 8–16 μg/mL. Notably, at 50 ppm, it achieved 100% inhibition against several key plant pathogenic fungi, such as *Fusarium graminearum* and *Rhizoctonia solani*, highlighting its potential utility in agricultural disease management. This potent bioactivity may represent a key ecological advantage for HMX112^T^ in its nutrient-poor desert environment. The primary antibacterial mechanism of thiolutin involves the inhibition of bacterial RNA polymerase, thereby blocking transcription (Qiu et al., 2024). Recent studies have also revealed its function as a zinc chelator, which, by inhibiting the JAMM metalloprotease Rpn11, disrupts the ubiquitin-proteasome system in eukaryotic cells, indicating potential antitumor activity and underscoring its multifunctional biological roles (Lauinger et al., 2017). Genomic analysis enabled the identification and functional assignment of the dedicated thiolutin biosynthetic gene cluster (*thi*), which not only genetically confirms the production capability of HMX112^T^ but also establishes a foundational platform for future yield optimization and structural derivatization via synthetic biology approaches. Interestingly, genomic analysis revealed that the identified biosynthetic gene cluster is phylogenetically closer to those for pyrroloformamides than to the canonical thiolutin cluster (Supplementary File 2), despite the isolation of thiolutin from fermentation. This suggests that the two clusters likely share a conserved core for synthesizing a common scaffold, with product specificity determined by strain-specific tailoring enzymes in the final biosynthetic steps (Zhou et al., 2020).

## Conclusion

This study successfully identified and characterized a novel bacterial species, *Streptomyces flavimicrosus* sp. nov. HMX112^T^, isolated from the Turpan-Hami Basin desert. Through comprehensive analysis, we confirmed its unique taxonomic status and demonstrated its ability to produce antimicrobial compounds. The strain’s genome was found to contain numerous biosynthetic gene clusters, indicating substantial potential for producing diverse natural products. We isolated and identified thiolutin as the key antibiotic compound from this strain. Laboratory tests confirmed that thiolutin effectively inhibits the growth of various pathogenic bacteria, including drug-resistant MRSA strains, as well as multiple plant pathogenic fungi. Furthermore, we identified the complete gene cluster responsible for thiolutin biosynthesis in HMX112^T^, providing genetic evidence for its production capacity. These findings establish *S. flavimicrosus* HMX112^T^ as a valuable microbial resource for antibiotic discovery. The combination of its novel taxonomic status, potent antimicrobial activity, and well-characterized biosynthetic pathways makes this strain particularly promising for future development of new anti-infective agents to address the challenge of antibiotic resistance.

In future studies, the functions of key genes can be validated through gene knockout, heterologous expression, and enzymatic assays, thereby elucidating the regulatory mechanisms underlying the biosynthesis of this compound and enabling systematic improvement of thiolutin production during fermentation. In addition, the potential synergistic effects of thiolutin in combination with other biopesticides or antibiotics warrant further investigation to evaluate its feasibility for combinational applications in agricultural and clinical settings.

### Description of *Streptomyces flavimicrosus* sp. nov.

*Streptomyces flavimicrosus* (fla.vi.mi.cro’sus; L. masc. adj. *flavus*, yellow; N.L. masc. adj. *microsus*, from Gr. masc. n. *mikros*, small, and L. v. *sus*, from *suscitare*, to arouse or excite; N.L. masc. adj. *flavimicrosus*, referring to a yellow-colored microbe that arouses interest due to its antimicrobial activity).

Aerobic, Gram-stain-positive actinobacterium that forms well-developed substrate and aerial mycelia. Yellow substrate mycelia and white aerial mycelia are observed on Gauze’s No. 1 medium, while pink colonies with filiform margins develop on yeast-starch agar. Smooth-surfaced, ovoid spores are borne in curved, ring-like spore chains. Growth occurs at 25–37°C (optimum 25–30°C), pH 5–11 (optimum pH 7–8), and in the presence of 0–5% (w/v) NaCl. Cell wall contains LL-diaminopimelic acid; whole-cell hydrolysates include ribose and glucose. Major menaquinones are MK-9(H8), MK-10(H4), and MK-9(H4). Polar lipids consist of diphosphatidylglycerol, phos phatidylglycerol, phosphatidylinositol, phosphotidylethanolamine, phosphatidylinositol mannosides, and several unidentified lipids. The predominant fatty acids are iso-C_16:0_, anteiso-C_15:0_, and anteiso-C_17:0_. The genome size is 6.52 Mbp with a G+C content of 72.93%. Produces the yellow antibiotic thiolutin, exhibiting broad-spectrum activity against pathogenic bacteria and fungi.

The type strain, HMX112^T^ (= GDMCC 4.392^T^ = CCM 9455^T^), was isolated from a desert soil sample collected from the Turpan-Hami Basin, Xinjiang, China. The GenBank accession numbers for the 16S rRNA gene sequence and whole-genome sequence of strain HMX112^T^ are PQ182579 and PRJNA1206132, respectively.

## Data Availability

The data presented in this study are publicly available. The data can be found at: https://www.ncbi.nlm.nih.gov/bioproject, accession PRJNA1206132.

## Footnotes

1
https://www.ezbiocloud.net/

2
https://orthovenn3.bioinfotoolkits.net
