# Supplementary figures and images for: A novel Streptomyces species producing thiolutin with anti-MRSA activity and insights into its biosynthetic gene cluster
Source: Front Microbiol. 2026 Feb 27;17:1759196. doi: 10.3389/fmicb.2026.1759196 (PMC12982422; doi:10.3389/fmicb.2026.1759196)

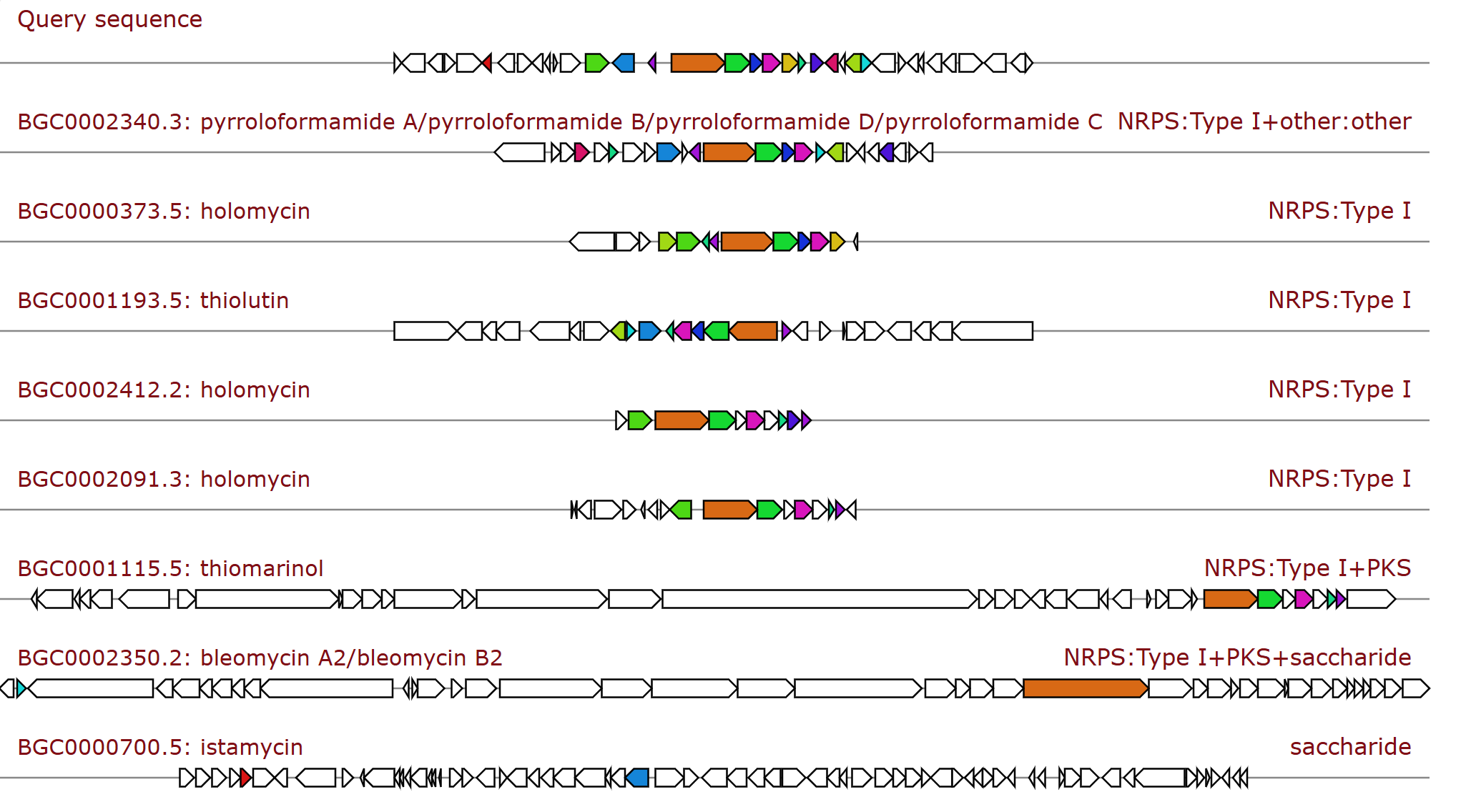


Figure 10 KnownClusterBlast analysis of the thiolutin biosynthetic gene cluster.

Supplement: Supplementary file 1 [file Supplementary_file_1.docx]
